# Caspase I-related protease inhibition retards the execution of okadaic acid- and camptothecin-induced apoptosis and PAI-2 cleavage, but not commitment to cell death in HL-60 cells

**DOI:** 10.1038/sj.bjc.6690269

**Published:** 1999-04

**Authors:** P H Jensen, K E Fladmark, B T Gjertsen, O K Vintermyr

**Affiliations:** Department of Medical Biochemistry, Aarhus University, Aarhus C, DK-8000, Denmark; Institute of Anatomy and Cell Biology, University of Bergen, Bergen, N-5009, Norway; Department of Pathology, Haukeland Hospital, University of Bergen, Bergen, N-5021, Norway

**Keywords:** okadaic acid, camptothecin, caspase 1, apoptosis, PAI-2, HL-60 cells

## Abstract

We have previously reported that the putative cytoprotective protease inhibitor, plasminogen activator inhibitor type 2 (PAI-2), is specifically cleaved during okadaic acid-induced apoptosis in a myeloid leukaemic cell line (*Br J Cancer* (1994) **70**: 834–840). HL-60 cells exposed to okadaic acid and camptothecin underwent morphological and biochemical changes typical of apoptosis, including internucleosomal DNA fragmentation and PAI-2 cleavage. Significant endogenous PAI-2 cleavage was observed 9 h after exposure to okadaic acid; thus correlating with other signs of macromolecular degradation, like internucleosomal DNA fragmentation. In camptothecin-treated cells, PAI-2 cleavage was an early event, detectable after 2 h of treatment, and preceding internucleosomal DNA fragmentation. The caspase I selective protease inhibitor, YVAD-cmk, inhibited internucleosomal DNA fragmentation and PAI-2 cleavage of okadaic acid and camptothecin-induced apoptotic cells. YVAD-cmk rather sensitively and non-toxically inhibited camptothecin-induced morphology, but not okadaic acid-induced morphology. In in vitro experiments recombinant PAI-2 was not found to be a substrate for caspase I. The results suggest that caspase I selective protease inhibition could antagonize parameters coupled to the execution phase of okadaic acid- and camptothecin-induced apoptosis, but not the commitment to cell death. © 1999 Cancer Research Campaign


*British Journal of Cancer* (1999) **79**(11/12), 1685–1691
© 1999 Cancer Research Campaign
Article no. bjoc.1998.0269

### Caspase I-related protease inhibition retards the execution of okadaic acid- and camptothecin-induced apoptosis and PAI-2 cleavage, but not commitment to cell death in HL-60 cells

**PH Jensen[1], KE Fladmark[2], BT Gjertsen[2] and OK Vintermyr[3]**

[1]Department of Medical Biochemistry, Aarhus University, DK-8000 Aarhus C, Denmark; [2]Institute of Anatomy and Cell Biology, University of Bergen, N-5009 Bergen, Norway; [3]Department of Pathology, Haukeland Hospital, University of Bergen, N-5021 Bergen, Norway

**Summary** We have previously reported that the putative cytoprotective protease inhibitor, plasminogen activator inhibitor type 2 (PAI-2), is specifically cleaved during okadaic acid-induced apoptosis in a myeloid leukaemic cell line (*Br J Cancer* (1994) **70**: 834–840). HL-60 cells exposed to okadaic acid and camptothecin underwent morphological and biochemical changes typical of apoptosis, including internucleosomal DNA fragmentation and PAI-2 cleavage. Significant endogenous PAI-2 cleavage was observed 9 h after exposure to okadaic acid; thus correlating with other signs of macromolecular degradation, like internucleosomal DNA fragmentation. In camptothecin-treated cells, PAI-2 cleavage was an early event, detectable after 2 h of treatment, and preceding internucleosomal DNA fragmentation. The caspase I selective protease inhibitor, YVAD-cmk, inhibited internucleosomal DNA fragmentation and PAI-2 cleavage of okadaic acid and camptothecin-induced apoptotic cells. YVAD-cmk rather sensitively and non-toxically inhibited camptothecin-induced morphology, but not okadaic acid-induced morphology. In in vitro experiments recombinant PAI-2 was not found to be a substrate for caspase I. The results suggest that caspase I selective protease inhibition could antagonize parameters coupled to the execution phase of okadaic acid- and camptothecin-induced apoptosis, but not the commitment to cell death.

**Keywords:** okadaic acid; camptothecin; caspase 1; apoptosis; PAI-2; HL-60 cells

Apoptosis is a fundamental biological process involved in embryogenesis, morphogenesis and tumour regression (Kerr et al, 1972; Wyllie et al, 1980). Recent observations suggest that modulation of protein phosphorylation (Gjertsen and Døskeland, 1995) and activation of cellular proteases are important factors regulating this type of cell death (reviewed in Zhivotovski et al, 1995; Golstein, 1997). The role of protein phosphorylation in regulation of apoptosis and activation of the downstream cascade of cystein proteases (caspases) is poorly understood. Inhibitors of serine/threonine protein phosphatases, like okadaic acid (OA), have proved useful to elucidate cellular functions regulated by phosphorylation (Cohen et al, 1990). OA, preferentially inhibiting type 2A protein phosphatases (Bialojan and Takai, 1988), induces swift and synchronous apoptotic cell death in various cell types (Bøe et al, 1991; Gjertsen et al, 1994; Kiguchi et al, 1994).

The caspase family of cell death proteases (Alnemri et al, 1996) is involved in the execution of apoptosis in mammalian cells (reviewed in Cohen, 1997). Less is known about their role in the triggering of apoptosis or whether this hierarchy of proteases may have separate functions in various types of cells. More specifically, the role of protein phosphorylation in regulation of apoptosis and activation of the downstream cascade of cystein proteases (caspases) is poorly understood. We have previously reported that the cytosolic protease inhibitor, plasminogen activator inhibitor type 2 (PAI-2), is specifically cleaved during OA-induced cell death in a promyelocytic leukaemic cell line and in myeloid leukaemic cells isolated from sternal bone marrow of patients suffering from leukaemia (Jensen et al, 1994). In fibrosarcoma cells and HeLa cells, transfected with PAI-2 expression vectors, PAI-2 was found to have a cytoprotective effect on tumour necrosis factor alpha (TNF-$\alpha$)-induced apoptosis (Kumar and Baglioni, 1991; Dickinson et al, 1995). In this regard PAI-2 is similar to the cowpox viral serine protease inhibitor CrmA, which abrogates apoptosis by inhibiting proteases of the caspase family (Ray et al, 1992) and granzyme B (Quan et al, 1995). However, PAI-2 reacts with Arg-specific proteases and not Asp-specific proteases like CrmA (Jensen, 1997). Recent observations, showing that PAI-2 interacts with cytosolic proteins through a domain localized between helices C and D (Jensen et al, 1996; Dickinson et al, 1998), suggest that not only its protease inhibitory function, but also its subcellular targeting could be important for its cellular function.

The aim of the present study was to test the role of protease activation during OA-induced apoptosis, and to define whether caspases are part of the proteolytic pathway leading to the apoptosis-associated PAI-2 cleavage. Secondly, to determine the event

*Received 20 May 1998*
*Revised 4 September 1998*
*Accepted 8 September 1998*

*Correspondence to:* OK Vintermyr of PAI-2 cleavage, in relation to other apoptosis-associated changes in morphology, nuclear chromatin structure, and internucleosomal DNA fragmentation. Camptothecin, a topoisomerase I inhibitor (reviewed in Rothenberg, 1997), was included in the study as an independent inducer of apoptosis (Del Bino et al, 1992; Shimizu and Pommier, 1997). We report that, although morphological and biochemical parameters of apoptosis, including PAI-2 cleavage, could be blocked by protease inhibition, the time course for commitment to cell death was not prolonged. The effect of protease inhibitors on OA and camptothecin-induced cell death, including PAI-2 cleavage, thus reflects more the inhibition of specific parts of the apoptotic process, e.g. the macromolecular breakdown, than the inhibition of cell death itself.

### MATERIALS AND METHODS

### Materials

Okadaic acid was from LC Services Corporation (Woburn, MA, USA). Camptothecin, dimethylsulphoxide (DMSO) and bisbenzimide (Hoechst 33342), were from Sigma (St Louis, MO, USA). YVAD (Ac-Tyr-Val-Ala-Asp-chloromethylketone) and Z-VAD fmk (*N*-benzyloxycarbony-Val-Ala-Asp (*O*-methyl)-fluoromethylketone were from Bachem Bioscience Inc. (Bubendorff, Switzerland). [$^{125}$I] (carrier free) was purchased from Amersham (Little Chalfont, UK). ECL immunoassay signal reagents and Hyperfilm MP were from Amersham (Little Chalfont, UK). We thank Drs Graeme Woodrow (Biotech Australia), Remy Sadoul (Glaxo Molecular Biology Laboratory, Geneva, Switzerland) and Winnie Wong (BASF Bioresearch Corporation, Worcester, MA, USA) for the kind gifts of recombinant human PAI-2, recombinant human proIL-1β and rabbit anti-proIL-1β serum and recombinant human ICE respectively.

### Cell culturing and handling of cells

The HL-60 cells were cultured in a I:I medium of RPMI and Dulbecco's modified Eagle's medium (DMEM; Sigma, St Louis, MO, USA) supplemented with 10% fetal calf serum (FCS; Biochrom KG, Germany), 2 mM glutamin, 100 IU ml$^{-1}$ penicillin and 100 μ ml$^{-1}$ streptomycin. In all experiments cells in logarithmic growth were seeded at a density of $4 \times 10^5$ per ml and kept below $10 \times 10^5$ per ml during the experimental period for optimal growth conditions. The various substances added to the cultures were dissolved in DMSO. The concentration of DMSO was routinely kept below 0.1% and shown not to interfere with cell survival. The cells were free of mycoplasma and other infections. In studies, aimed at testing the commitment to cell death after exposure to various substances, cell aliquots were quenched in 10 volumes of culture medium, spun ($700 \times g_{av.}$ for 5 min), and washed twice in complete medium before reseeding, ensuring a theoretical 3000-fold dilution of added substances.

### Nuclear chromatin condensation assay

Aliquots of variously treated HL-60 cells were fixed in 2% glutaraldehyde in 0.1 M Na-cacodylate buffer (pH 7.4) supplemented with the DNA-specific fluorochrome, bisbenzimide. The nuclear chromatin condensation was determined as previously described (Bøe et al, 1995). Normal (non-apoptotic) cells had a uniform vague nuclear fluorescence, whereas apoptotic cells showed an increased fluorescence from condensed non-fragmented (OA-treated cells) or fragmented (camptothecin-treated cells) nuclei; the latter were easily distinguished from mitotic cells. Few cells emitted an intermediate fluorescence pattern. Normal and apoptotic cells were found to exclude trypan blue (0.2%), in contrast to necrotic cells.

### Electron microscopy

HL-60 cells ($3 \times 10^5$ cells) were fixed at 37°C in 0.1 M Na-cacodylate buffer, pH 7.4 containing 2% glutaraldehyde and placed on ice for 15 min. The samples were subsequently rinsed three times in 0.1 M Na-cacodylate buffer and post-fixed in buffer containing 1% $OsO_4$. The cells were then dehydrated, embedded in resin, sectioned and stained with uranyl acetate and lead citrate as previously described (Bøe et al, 1991). The specimens were examined in a Jeol 100CX electron microscope. The microphotographs were digitalized using an AGFA Arcus II scanner and processed by Adobe Photoshop software.

### DNA-fragmentation assay

Cell pellets containing $3 \times 10^6$ cells were dissolved in 0.5 ml cell lysis buffer (100 mM EDTA, 10 mM EGTA, 0.5% sodium dodecyl sulphate (SDS), 10 mM Tris-HCl, pH 8.0), and subsequently treated with 30 μg ml$^{-1}$ RNAase and 100 μg ml$^{-1}$ protease K, extracted in 10 mM Tris-buffered phenol, pH 8.0, washed twice in 70% ethanol, air dried and re-dissolved in 10 mM Tris-HCl, 1 mM EDTA, pH 7.5 as described elsewhere (Gjertsen et al, 1994). DNA aliquots (10 μg) were electrophoresed in 1.5% agarose gels and visualized under UV illumination after staining with 0.5 μg ml$^{-1}$ ethidium bromide. The ratio of high molecular weight (HMW) DNA (above 8.5 kb) versus low molecular weight (LMW) DNA (0.1–7.2 kb) was determined by densiometric measurement of the negative Polaroid 665 film using a LKB Ultroscan XL laser densiometer as described previously (Gjertsen et al, 1994).

### In vivo PAI-2 cleavage

For determination of PAI-2 cleavage $2 \times 10^6$ cells were centrifuged ($1000 \times g_{av.}$ for 5 min) and dissolved in PAI-2 lysis buffer (120 mM NaCl, 50 mM HePes, pH 7.4, 5 mM EDTA, 3 mM EGTA, 0.05 mg ml$^{-1}$ aprotinin, 1 mg ml$^{-1}$ soybean trypsin inhibitor, 2 mM phenylmethane-sulphonylfluoride, 0.5 mM dithioerythritol, 250 mM sucrose, 1% Triton X-100 (all from Sigma, St. Louis, MO, USA, except when noted) at a concentration of approximately 1.5 $\times 10^7$ per ml. The lysate was homogenized by three strokes for 5 s in an Ultra Turax homogenizer, aliquoted and snap frozen in liquid nitrogen. The samples were stored at – 80°C. Cell extracts corresponding to $0.5 \times 10^6$ cells were loaded in each slot of a reducing 8–16% gradient SDS-polyacrylamide gel (PAGE) (Jensen et al, 1990), resolved and electroblotted onto polyvinylidene fluoride membranes. The residual protein-binding capacity was quenched by 2% skimmed milk, 0.5% Tween-20 in phosphate-buffered saline (PBS) (quenching buffer) and probed with the monoclonal anti-PAI-2 antibody (#3750, Amersham Diagnostics) diluted 1/350 in quenching buffer. Bound antibody was detected using the ECL immunoassay signal reagents and Hyperfilm MP.

### In vitro ICE-cleavage assay

Recombinant human IL-1β (30 μg ml$^{-1}$) and PAI-2 (60 μg ml$^{-1}$) were incubated in the absence or presence of recombinant human

 © Cancer Research Campaign 1999

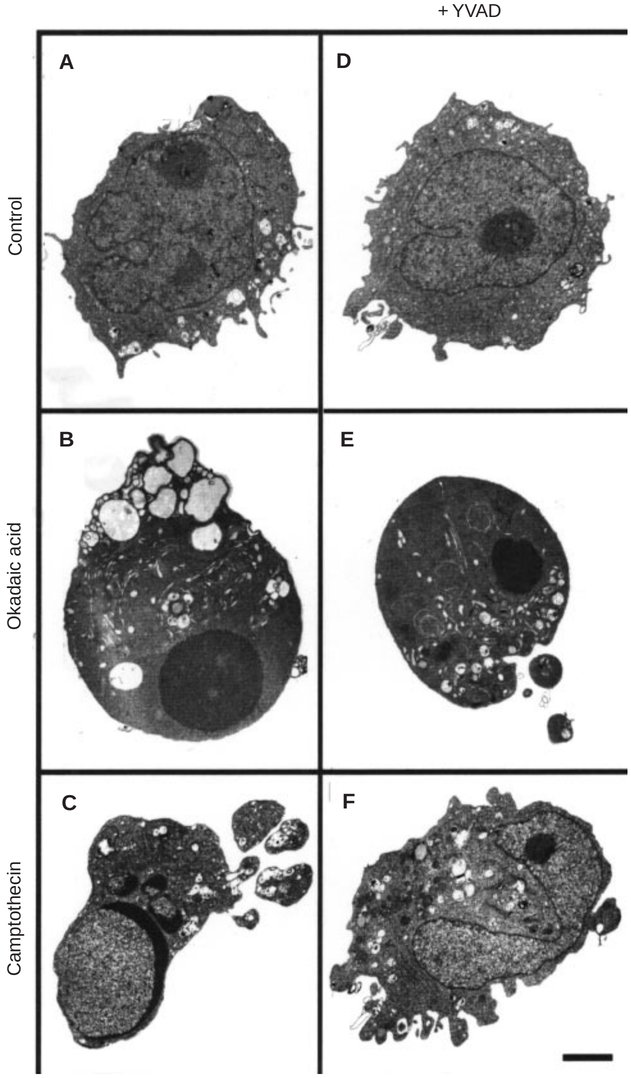

**Figure 1** The phenotypic apoptotic morphologies induced by okadaic acid or camptothecin in HL-60 cells are sensitive to inhibition by YVAD-cmk. The cells were cultured in absence (**A**, **D**) or presence of 300 nM OA (OA) for 6 h (**B**, **E**) or 5 μM camptothecin for 4 h (**C**, **F**). Some of the cells were supplemented with 300 μM YVAD-cmk (**D**, **E**, **F**). Micrographs show representative morphology of the variously treated cells. The bar in panel F represents 1 μm

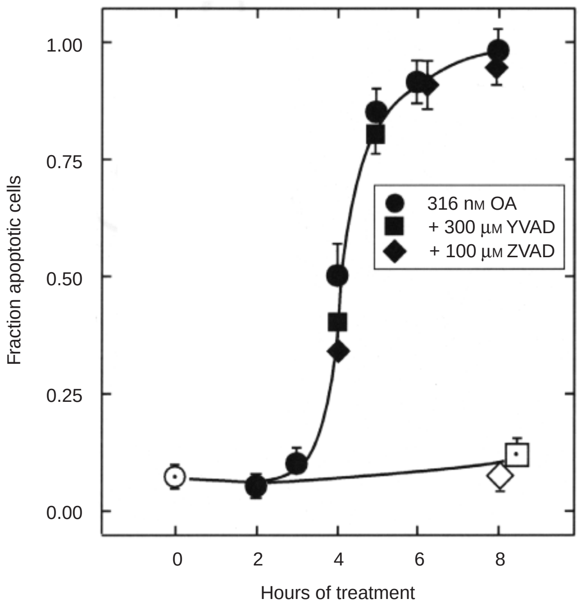

**Figure 2** OA-induced apoptotic nuclear chromatin condensation is not affected by YVAD-cmk or Z-VAD-fmk. The cells were treated with 300 nM OA (•), 300 nM OA and 300 μM YVAD-cmk (■), 300 nM OA and 100 μM Z-VAD-fmk (◆), 300 μM YVAD-cmk (□), 100 μM Z-VAD-fmk (◇), or left unsupplemented as control (○). At regular time points cell aliquots were fixed and stained with bisbenzimide, and the fraction of cells that had condensed nuclear chromatin was determined. The bars show standard error of the mean from three or more separate experiments

ICE (3 μg ml$^{-1}$) for 6 h in 100 mM Hepes, pH 7.5, 5 mM dithioerythritol, 0.5 mM EDTA, 20% glycerol. The samples were added equal volumes 2 × reducing SDS-electrophoresis sample buffer and resolved by 10–20 gradient SDS-PAGE. The gel was analysed by immunoblotting as described above except for the detection of interleukin (IL)-1β, which was performed using rabbit anti-human IL-1β diluted 1/500. The bound antibodies were detected as mentioned above.

### RESULTS

### Apoptosis induced by okadaic acid and camptothecin differs phenotypically in HL-60 cells

OA-induced cell death in HL-60 cells was characterized by an early loss of microvilli, pronounced blebbing of the plasma membrane, cytosolic vacuolization beneath the plasma membrane and segregation of subcellular organelles forming interconnected clusters of membrane-bound organelles (Figure 1B). The major cytosolic effects rather closely paralleled the onset of nuclear chromatin condensation. The time course for the transition from apparent normal (non-condensed) to markedly condensed nuclear chromatin was swift, rendering few cells in the transitional state. Exposure of HL-60 cells to 300 nM OA-induced nuclear chromatin condensation in more than 90% of the cells within 6 h (Figure 2) strongly favouring this process to be independent of cell cycle phase. On exposure to lower OA concentration cell death was induced less synchronously; thus only half the cells were morphologically affected after challenging with 100 nM OA for 12 h. Apoptosis induced by camptothecin occurred more swiftly, but less synchronously, than with OA and was characterized with a pronounced formation of membrane-encapsulated apoptotic bodies and extensive nuclear fragmentation (Figure 1C, Figure 3). All early phase apoptotic cells excluded trypan blue suggesting that the integrity of the plasma membranes remained intact in these cells.

### Okadaic acid- and camptothecin-induced apoptosis is sensitive to protease inhibition by YVAD-cmk and Z-VAD-fmk but differs phenotypically

The peptide inhibitors, YVAD-cmk and Z-VAD-fmk, known to affect the caspase cascade of proteases involved in apoptosis, were tested for their ability to modulate OA- and camptothecin-induced apoptosis. YVAD-cmk and Z-VAD-fmk did not markedly affect cytosolic or nuclear morphological effects of apoptosis induced by OA (Figure 1E), although the nuclear chromatin condensation was less pronounced and more patchy in the early apoptotic phase. The

© Cancer Research Campaign 1999

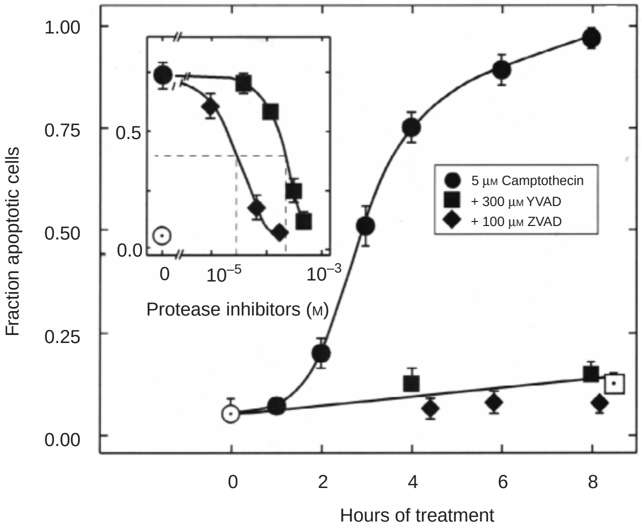

**Figure 3** Camptothecin-induced apoptotic nuclear chromatin condensation is sensitive to inhibition by YVAD-cmk or Z-VAD-fmk. The cells were treated with 5 µM camptothecin (•), 5 µM camptothecin and 300 µM YVAD-cmk (■), 5 µM camptothecin and 100 µM Z-VAD-fmk (◆), 300 µM YVAD-cmk (□), 100 µM Z-VAD-fmk (◇), or left unsupplemented as control (○). At regular time points cell aliquots were fixed and stained with bisbenzimide, and the fraction of cells that had condensed nuclear chromatin was determined. The bars show standard error of the mean from 3–8 separate experiments. **Inset**: Potency of protease inhibitors to inhibit camptothecin-induced nuclear chromatin condensation. The cells were treated with 5 µM camptothecin in absence (•) or presence of varying concentrations of YVAD-cmk (■), Z-VAD-fmk (◆), or left unsupplemented as control (○). After treatment for 4 h, cell aliquots were fixed and stained with bisbenzimide. The bars show standard error of the mean from 3–5 separate experiments peptide protease inhibitors were found to counteract all morphological features of camptothecin-induced apoptosis including condensation and fragmentation of nuclear chromatin (Figure 1F).

Specific degradation of chromosomal DNA was a prominent feature of both OA- and camptothecin-induced cell death (Figure 4). YVAD-cmk counteracted the action of both agents rendering a greater fraction of non-degraded chromosomal DNA, although the internucleosomal DNA cleavage was not completely blocked. YVAD-cmk did not induce spontaneous internucleosomal DNA fragmentation at the concentrations used in the study (data not shown).

Immunoblotting of cytosolic PAI-2 detected a 33 kDa PAI-2 fragment (Figure 5) with retained protease inhibition activity (data not shown) during OA- and camptothecin-induced apoptosis in HL-60 cells. The cleavage of PAI-2 during camptothecin-induced apoptosis was an early event being detectable after 2 h of treatment (Figure 5, lane 3). The PAI-2 cleavage induced by OA and camptothecin was sensitive to the caspase I inhibitor YVAD-cmk (Figure 5, lanes 5 and 7). As YVAD-cmk is an efficient inhibitor of IL-1β-converting enzyme (ICE), we tested whether PAI-2 was a substrate for ICE. In an in vitro assay, using pro-IL-1β as a positive control, recombinant PAI-2 was not found to be a substrate for ICE (Figure 6, lanes 3 and 4).

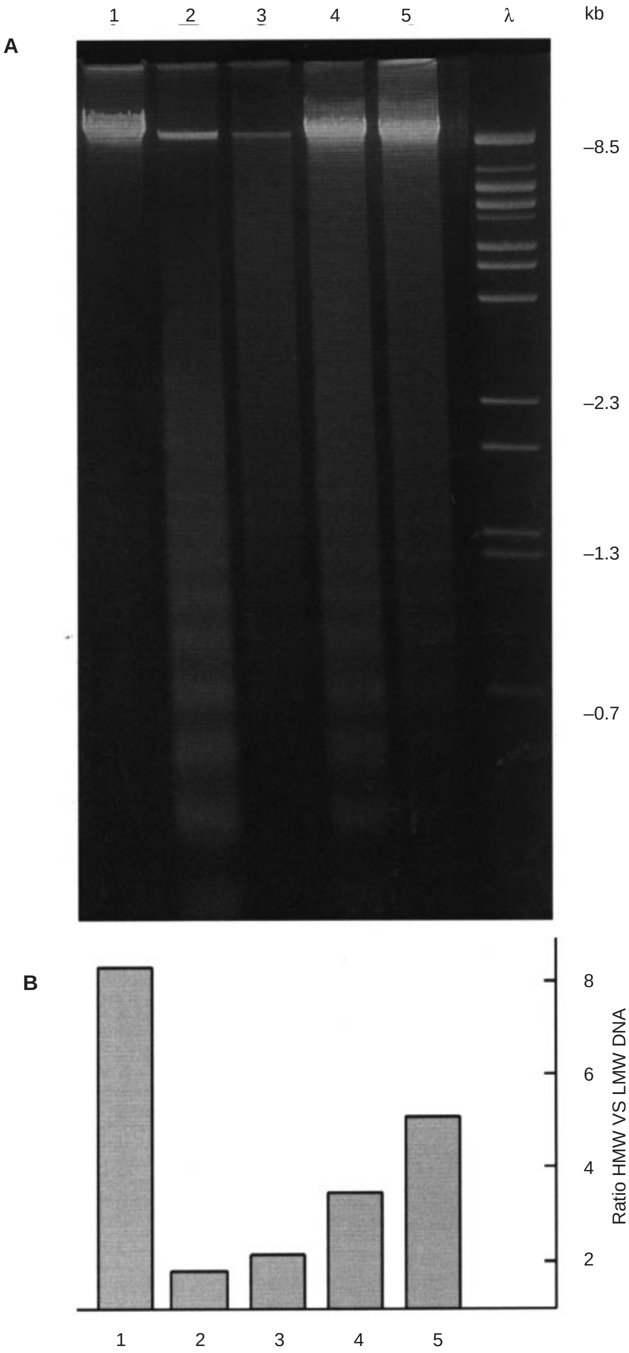

**Figure 4** (**A**) Inhibition of OA- and camptothecin-induced internucleosomal DNA fragmentation by YVAD-cmk. The cells were treated with 5 µM camptothecin for 6 h (lanes 2 and 4) or 300 nM OA for 9 h (lanes 3 and 5). The cultures shown in lanes 4 and 5 were supplemented with 300 µM YVAD-cmk. Lane 1 represents control cells after 9 h. The size of the internucleosomal DNA fragments was in the range 195–200 base pairs as evidenced by the λ-DNA reference marker (lane λ). The extracted DNA was subjected to agarose gel electrophoresis and the results from one representative experiment shown. (**B**) The ratio of HMW DNA (above 8.5 kb) versus LMW DNA (0.1–7.2 kb) was determined for each line 1–5 above as outlined in Methods section

### OA- and camptothecin-induced commitment to cell death is not retarded by YVAD-cmk

Induction of apoptosis is thought to proceed through a series of steps, initially reversible, but inevitably becoming irreversible leading to cell death. The time course for commitment to cell death in HL-60 cells treated with OA or camptothecin was assessed by determining the fraction surviving cells having normal morphology 24 h after short periods of exposure to these agents

© Cancer Research Campaign 1999

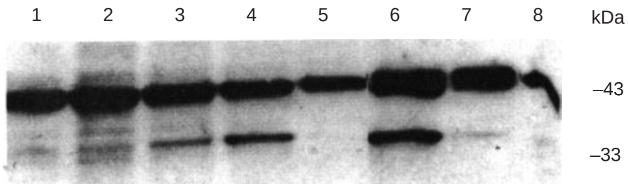

**Figure 5** Inhibition of OA- and camptothecin-induced selective PAI-2 cleavage by YVAD-cmk. Cells ($0.5 \times 10^6$ per ml) were incubated in the presence of 5 µM camptothecin for 1, 2, and 4 h respectively (lanes 2–5) or 300 nM OA for 9 h (lanes 6,7). Lane 1 represents untreated (control) cells and lanes 5, 7 and 8 cells co-incubated with 300 µM YVAD-cmk. The cells were extracted and processed by reducing 8–16% gradient SDS-PAGE, anti-PAI-2 immunoblotting and ECL visualization as described in Methods section. The estimated molecular weights, 43 kDa for native PAI-2 and 33 kDa for the PAI-2 cleavage product, respectively, are indicated

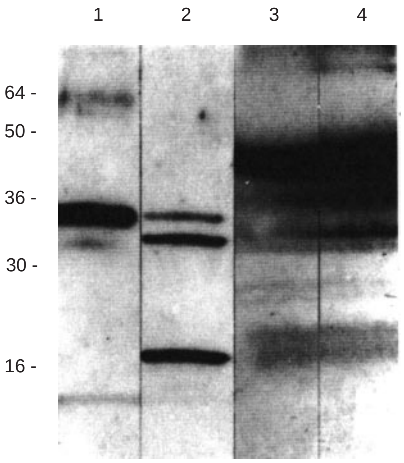

**Figure 6** Interleukin-1β-converting enzyme (ICE) does not cleave PAI-2 in vitro. Recombinant human pro-IL-1β (30 µg $ml^{-1}$; lanes 1–2) and recombinant human PAI-2 (60 µg $ml^{-1}$; lanes 3–4) were incubated in the absence (lanes 1, 3) or presence (lanes 2, 4) of recombinant ICE (3 µg $ml^{-1}$) for 6 h. The samples were processed by reducing 10–20% gradient SDS-PAGE, anti-IL-1β immunoblotting and ECL detection as described in Methods section. The molecular weight markers are indicated to the left

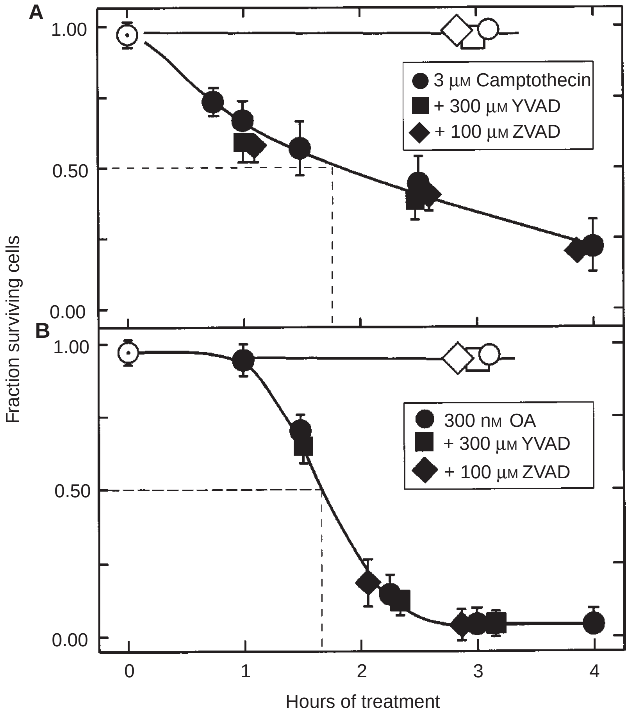

**Figure 7** The time course for commitment to apoptosis induced by okadaic acid or camtothecin is not prolonged by protease peptide inhibitors. (**A**). The cells were treated with 5 µM camptothecin (•), 5 µM camptothecin and 300 µM YVAD-cmk (■), 5 µM camptothecin and 100 µM Z-VAD-fmk (◆), 300 µM YVAD-cmk (□), 100 µM Z-VAD-fmk (◇), or left unsupplemented as controls (○). (**B**) The cells were treated with 300 µM OA (•), 300 µM OA and 300 µM YVAD-cmk (■), 300 nM OA and 100 µM Z-VAD-fmk (◆), 300 µM YVAD-cmk (□), 100 µM Z-VAD-fmk (◇), or left unsupplemented as controls (○). At times indicated, cell aliquots were extensively washed (see Methods section), reseeded, and cultured for the remaining time of a 24-h period. After 24 h of culture the fraction of surviving cells was determined as the fraction of cells having an intact (normal) non-condensed nuclear chromatin appearance. The dying (apoptotic) cell fractions all show condensed and/or fragmented nuclear chromatin. The bars show standard error of the mean from 3–5 separate experiments

(see Methods section for details). OA (300 nM) induced a swift commitment to cell death; more than 50% of the cells were doomed to death after 1.75 h of treatment. The commitment to cell death by OA displayed a lag phase of about 1 h. During this time period OA could be washed away without affecting the long-term survival of the cells (Figure 7). No apparent time lag was noted for camtothecin-induced apoptosis and the time course for commitment to cell death was more protracted (Figure 7).

YVAD-cmk (300 µM) or Z-VAD-fmk (100 µM) did not affect long-term survival of HL-60 cells (Figure 7). Although YVAD-cmk (or Z-VAD-fmk) counteracted morphological effects of apoptosis, inhibited internucleosomal DNA fragmentation and abolished PAI-2 cleavage, the commitment to cell death by OA and camptothecin was not retarded by co-treatment with YVAD-cmk (Figure 7).

### DISCUSSION

Our data show that okadaic acid and camptothecin induce apoptosis with different morphological phenotypes (Figure 1). That one single cell type can undergo different apoptotic phenotypes has previously been shown in experimental studies (Gjertsen et al, 1994) and in studies on developmental cell death (Clarke, 1990). Together these observations suggest that different regulatory mechanisms are related to distinct cell death phenotypes. From a therapeutic point of view this could be favourably, since resistance against apoptosis involving one pathway, e.g. from loss of certain caspase(s), can be overcome by triggering of apoptosis through alternative pathways not dependent of the lacking caspase(s).

The role of caspase I in apoptosis is controversial (Nicholson and Thornberry, 1997). Mice deficient in IL-1β (caspase I) develop normally, suggesting a less important role of caspase I in regulation of cell death during normal embryogenesis (Kuida et al, 1995). On the other hand, thymocytes from IL-1β-deficient mice were found to be resistant to *Fas*-induced apoptosis showing an impairment of the normal regulation of apoptosis in these cells (Kuida et al, 1995). We report that YVAD-cmk, a potent inhibitor of caspase I protease activity (Schumann et al, 1998), inhibits both OA- and camptothecin-induced internucleosomal DNA degradation (Figure 4) and PAI-2 cleavage (Figure 5). However, only the

© Cancer Research Campaign 1999

phenotypic apoptotic morphology induced by camptothecin was antagonized by YVAD-cmk and by Z-VAD-fmk (Figure 1–3), the latter being a more broadly acting caspase inhibitor. The inhibition of camptothecin-induced apoptosis by YVAD-cmk was observed at concentrations above 30 μM and with a 50% inhibitory concentration ($IC_{50}$) of about 210 μM (Figure 3, inset). Knowing that the inhibition constant for caspase I by YVAD-CHO is several orders of magnitude lower than for inhibition of caspase 3 (Nicholson and Thornberry, 1997) and that experiments in vitro have shown $IC_{50}$ for inhibition of IL-1β in the low μM range (Kuida et al, 1995), our results in vivo thus point to a role for a caspase I-related activity in the execution phase of camptothecin and of OA-mediated apoptosis in HL-60 cells.

A key object of this study has been to test whether caspase inhibitors could modulate the time course for initiation (commitment) of apoptosis by OA and camptothecin. The results were conclusive on this point. Neither YVAD-cmk nor Z-VAD-fmk were able to modulate the commitment to cell death by OA or camptothecin. Additionally, treatment with YVAD-cmk or Z-VAD-fmk had no adverse effect on HL-60 cell survival. Our results are thus compatible with results in rat-1 fibroblast in which the inhibition of ced-3/ICE-related proteases could not prevent cell death induced by oncogenes, DNA damage or bak (a bcl-2 homologue) although the execution phase of apoptosis was inhibited (McCarthy et al, 1997). OA-treated cells showed a lag phase of about 1 h during which time period the cells were fully non-committed to cell death after removal of the drug (Figure 6). This reversible lag period, corresponding to the induction time or lag phase of apoptosis (Jakobson et al, 1994; Greenlund et al, 1995) preparing the signal transduction machinery for cell suicide, was not sensitive to caspase peptide inhibition. This observation supports the idea that signalling pathways other than the cell death proteases could be important for the regulation of the commitment phase of OA-induced apoptosis. This is also supported by studies in primary hepatocytes in which effects of serine/threonine protein phosphatase inhibitors, including the induction of apoptosis, are reversibly inhibited by antagonists of $Ca^{2+}$/calmodulin-dependent protein kinase type II (Mellgren et al, 1997; Toivola et al, 1997, and unpublished observations).

In a previous communication we showed that PAI-2 was specifically cleaved during OA-induced apoptotic cell death in a promyelocytic leukaemic cell line (Jensen et al, 1994). This was, to our knowledge, the first demonstration of selective proteolytic cleavage of a putative cytoprotective cellular protease inhibitor (Kumar and Baglioni, 1991). The time course for this cleavage, whether associated with the induction/commitment or the execution phases of apoptotic cell death, has been tested in this study. In OA-treated cells the time course for PAI-2 cleavage was associated with internucleosomal DNA degradation thus being linked to the macromolecular degradation typical of the execution phase of apoptosis (Figures 4 and 5). In camptothecin-treated cells the cleavage of PAI-2 was an early event that accompanied other early morphological effects of apoptosis like the membrane blebbing and thus preceded the macromolecular degradation of DNA by several hours. The cause for this difference in the timing of PAI-2 cleavage is not clear, but is supportive of the idea that OA- and camptothecin-induced apoptosis involve partly divergent proteolytic pathways. Also, whether this difference in time course for the PAI-2 cleavage represents a cell-specific phenomenon or not, remains to be clarified. PAI-2 was not found to be a substrate for recombinant IL-1β (caspase 1) in vitro (Figure 6) although YVAD-cmk inhibited camptothecin and OA mediated PAI-2 selective cleavage in cells, and prevented caspase I-mediated cleavage of pro-IL-1β in vitro (Figure 6). This led us to suggest that the PAI-2 cleavage should be mediated by some other apoptosis-associated protease.

We report that the execution phase of OA- and camptothecin-induced apoptosis in HL-60 cells was sensitive to protease inhibitors selecting caspase I whereas the induction or commitment phase was not. Our data suggest that diverse proteolytic mechanisms are involved in the execution phase of OA and camptothecin, some being sensitive to caspase I-related inhibition, others not. Moreover, the results are focused on the degradation of PAI-2, a putative cytoprotective protease inhibitor, in relation to the commitment and execution phases of OA- and camptothecin-induced apoptosis in HL-60 cells.

### ACKNOWLEDGEMENTS

The skilled technical assistance of Lis Hygum, Erna Finsås and Berit Hausvik is highly appreciated. This study was supported by the Norwegian Cancer Society, Danish Cancer Society, EU Biomedical Programme, Michaelis Fonden and Familien Blix fund.

### REFERENCES

Alnemri ES, Livingston DJ, Nicholson DW, Salvesen G, Thornberry NA, Wong WW and Yuan J (1996) Human ICE/CED-3 protease nomenclature. *Cell* **87**: 171

Bialojan C and Takai A (1988) Inhibitory effect of a marine sponge toxin, okadaic acid, on protein phosphatases. *Biochem J* **256**: 283–290

Bøe R, Gjertsen BT, Vintermyr OK, Houge G, Lanotte M and Døskeland SO (1991) The protein phosphatase inhibitor okadaic acid induces morphological changes typical of apoptosis in mammalian cells. *Exp Cell Res* **195**: 237–246

Bøe R, Gjertsen BT, Døskeland SO and Vintermyr OK (1995) 8-CI-cAMP triggers apoptosis in human mammary carcinoma cells (MCF-7) independently of the cAMP kinase. *Br J Cancer* **72**: 1151–1159

Cohen GM (1997) Caspases: the executioners of apoptosis. *Biochem J* **326**: 1–16

Cohen P, Holmes CFB and Tsukitani Y (1990) Okadaic acid: a new probe for the study of cellular regulation. *TIBS* **15**: 98–102

Clarke PG (1990) Developmental cell death: morphological diversity and multiple mechanisms. *Anat Embryol* **181**: 195–213

Del Bino G, Bruno S, Yi PN and Darzynkiewicz Z (1992) Apoptotic cell death triggered by camptothecin or teniposide. The cell cycle specificity and effects of ionizing radiation. *Cell Prolif* **25**: 537–548

Dickinson JL, Bates EJ, Ferrante A and Antalis TM (1995) Plasminogen activator inhibitor type 2 inhibits tumour necrosis factor alpha-induced apoptosis. Evidence for an alternate biological function. *J Biol Chem* **270**: 27894–27904

Dickinson JL, Norris BJ, Jensen PH and Antalis TM (1998) The CD-interhelical domain of the serpin plasminogen activator inhibitor-type 2 is required for protection from TNFalfa induced apoptosis. *Cell Death Diff* **5**: 163–171

Gjertsen BT and Døskeland SO (1995) Protein phosphorylation in apoptosis. *Biochim Biophys Acta* **1269**: 187–199

Gjertsen BT, Cressey LI, Ruchaud S, Houge G, Lanotte M and Døskeland SO (1994) Multiple apoptotic death types triggered through activation of separate pathways by cAMP and inhibitors of protein phosphatases in one (IPC leukemia) cell line. *J Cell Sci* **107**: 3363–3377

Golstein P (1997) Controlling cell death. *Science* **275**: 1081–1082

Greenlund LJS, Korsmeyer SJ and Johnson EM Jr (1995) Role of bcl-2 in the survival and function of developing and mature sympathetic neurons. *Neuron* **15**: 649–661

Jacobson MI, Burne JF and Raff MC (1994) Programmed cell death and bcl-2 protection in the absence of a nucleus. *EMBO J* **13**: 1899–1910

Jensen PH (1997) Structure and function of plasminogen activator inhibitor-2: an intracellular serine proteinase inhibitor modulating apoptosis. *Int J Oncol* **11**: 557–570

 © Cancer Research Campaign 1999

Jensen PH, Christensen EI, Ebbesen P, Gliemann J and Andreasen PA (1990) Lysosomal degradation of receptor-bound urokinase-type plasminogen activator is enhanced by its inhibitors in human trophoblastic choriocarcinoma cells. *Cell Reg* **1**: 1043–1056

Jensen PH, Cressey LI, Gjertsen BT, Madsen P, Mellgren G, Hokland P, Gliemann J, Døskeland SO, Lanotte M and Vintermyr OK (1994) Cleaved intracellular plasminogen activator inhibitor-2 in human myeloleukemia cells is a marker of apoptosis. *Br J Cancer* **70**: 834–840

Jensen PH, Jensen TG, Laug WE, Hager H, Gliemann J and Pepinsky B (1996) The exon 3 encoded sequence of the intracellular serine proteinase inhibitor plasminogen activator inhibitor 2 is a protein binding domain. *J Biol Chem* **271**: 26892–26899

Kerr JFR, Wyllie AH and Currie AR (1972) Apoptosis: a basic biological phenomenon with wide-ranging implications in tissue kinetics. *Br J Cancer* **26**: 239–257

Kiguchi K, Glesne D, Chubb CH, Fujiki H and Hubermann E (1994) Differential induction of apoptosis in human breast tumour cells by okadaic acid and related inhibitors of protein phosphatases 1 and 2A. *Cell Growth Diff* **5**: 995–1004

Kuida K, Lippke JA, Ku G, Harding MW, Livingston DJ, Su MSS and Flavell RA (1995) Altered cytokine export and apoptosis in mice deficient in interleukin-1β converting enzyme. *Nature* **267**: 1998–2002

Kumar S and Baglioni C (1991) Protection from tumour necrosis factor-mediated cytolysis by overexpression of plasminogen activator inhibitor type-2. *J Biol Chem* **266**: 20960–20964

McCarthy NJ, Whyte MKB, Gilbert CS and Evan GI (1997) Inhibition of Ced-3/ICE-related proteases does not prevent cell death induced by oncogenes, DNA damage, or the bcl-2 homologue bak. *J Cell Biol* **136**: 215–227

Mellgren G, Bruland T, Døskeland AP, Flatmark T, Vintermyr OK and Døskeland SO (1997) Synergistic antiproliferative actions of cyclic adenosine 3′,5′-monophosphate, interleukin-1β, and activators of Ca++/calmodulin-dependent protein kinase in primary hepatocytes. *Endocrinology* **138**: 4373–4383

Nicholson DW and Thornberry NA (1997) Caspases: killer proteases. *TIBS* **22**: 299–306

Quan LT, Caputo A, Bleackley RC, Pickup DJ and Salvesen GS (1995) Granzyme B is inhibited by the cowpox virus serpin cytokine response modifier. *J Biol Chem* **270**: 10377–10379

Ray CA, Black RA, Kronheim SR, Greenstreet TA, Sleath PR, Salvesen GS and Pickup DJ (1992) Viral inhibition of inflammation: cowpox virus encodes an inhibitor of the interleukin-1-beta converting enzyme. *Cell* **69**: 597–604

Rothenberg ML (1997) Topoisomerase I inhibitors: review and update. *Ann Oncol* **8**: 837–855

Schumann RR, Belka C, Reuter D, Lamping N, Kirschning CJ, Weber JR and Pfeil D (1998) Lipopolysaccharide activates caspase-1 (interleukin-1-converting enzyme) in cultured monocytic and endothelial cells. *Blood* **15**: 577–584

Shimizu T and Pommier Y (1997) Camptothecin-induced apoptosis in p53-null human leukemia HL-60 cells and their isolated nuclei: effects of the protease inhibitors Z-VAD-fmk and dichloroisocoumarin suggest an involvement of both caspases and serine proteases. *Leukemia* **11**: 1238–1244

Toivola DM, Goldman RD, Garrod DR and Eriksson JE (1997) Protein phosphatases maintain the organization and structural interactions of hepatic keratin intermediate filaments. *J Cell Sci* **110**: 23–33

Wyllie AH, Kerr JF and Currie AR (1980) Cell death: The significance of apoptosis. *Int Rev Cytol* **68**: 251–305

Zhivotovski B, Gahm A, Ankarcrona M, Nicotera P and Orrenius S (1995) Multiple proteases are involved in thymocyte apoptosis. *Exp Cell Res* **221**: 404–412

*© Cancer Research Campaign 1999*
